# Epidemiological characteristics of severe fever with thrombocytopenia syndrome and the relationship with meteorological factors in Jiangsu Province, China

**DOI:** 10.3389/fpubh.2025.1662670

**Published:** 2025-09-01

**Authors:** Yuhao Tao, Wendong Liu, Yin Wang, Shuyi Liang, Xiaochen Wang, Zhifeng Li, Wenxin Gu, Xin Liu, Xinru Zeng, Changjun Bao

**Affiliations:** ^1^School of Public Health, Nanjing Medical University, Nanjing, China; ^2^Jiangsu Provincial Center for Disease Control and Prevention, Nanjing, China; ^3^Department of Acute Infectious Diseases Control and Prevention, Yangzhou Center for Disease Control and Prevention, Yangzhou, China

**Keywords:** severe fever with thrombocytopenia syndrome, epidemiological characteristics, meteorological factors, temperature, distributed lag non-linear model

## Abstract

**Background:**

Severe Fever with Thrombocytopenia Syndrome (SFTS) has become a global public health concern in recent years. The main purpose of this study was to depict the epidemiological characteristics of SFTS in Jiangsu Province, China and evaluate the effects of meteorological factors on its dynamics.

**Methods:**

Joinpoint regression and spatial methods were used to explore the epidemiological trends of SFTS. Distributed Lag Non-linear Models (DLNM) were employed to evaluate the regional specific effects of meteorological factors on SFTS epidemics.

**Results:**

Between 2014 and 2023, a total of 914 SFTS cases were reported in Jiangsu Province, with an average annual incidence of 0.11 per 100,000 population and an overall case fatality rate of 7.44%. Cases aged 50–79 years accounted for 83.48% (763/914) of the total cases. Most affected individuals were farmers (69.14%, 632/914). Joinpoint regression analysis revealed an increasing trend in the incidence rate of SFTS, with an Annual Percent Change of 26.68% (*p* < 0.001). The incidence presented marked seasonality, with the peak occurring between May and July. Nanjing and Huai’an were the major endemic regions, together accounting for 79.32% of the total cases in the province. DLNM results showed an inverted “V”-shaped relationship between weekly average temperature and the risk of SFTS incidence in Nanjing and Huai’an, with temperature inflection points of 22.4 °C and 19.2 °C, respectively. In both cities, lower temperatures (Nanjing: 3.35, 8.77 °C; Huai’an: 1.73, 7.15 °C) were associated with a reduced risk of SFTS incidence, whereas higher temperatures (Nanjing: 24.32, 28.05 °C; Huai’an: 23.89, 27.15 °C) were associated with an increased risk. However, excessively high temperatures can also lead to a decrease in the incidence of SFTS. Targeted strategies and measures should be taken to prevent its spread.

**Conclusion:**

In conclusion, over the study period, SFTS incidence and geographic spread in Jiangsu Province showed an increasing trend. Temperature exerted a non-linear impact on the incidence of SFTS, characterized by notable lag effects.

## 1 Introduction

Severe fever with thrombocytopenia syndrome (SFTS) is an emerging zoonotic infectious disease attributed to the Severe fever with thrombocytopenia syndrome virus (SFTSV), first identified in rural China in 2009 (1, 2). Ticks, serving as the primary vectors, play a crucial role in the transmission of SFTS (3, 4). The disease is primarily transmitted via bites from ticks carrying the pathogen. Some studies indicate that exposure to the blood or secretions of confirmed patients, as well as contact with the blood or tissues of infected animals (e.g., goats, cattle, or dogs), poses a risk of human infection (5, 6). Common symptoms following infection include fever, thrombocytopenia, leukopenia, and gastrointestinal symptoms (7, 8). In severe cases, patients may suffer multi-organ damage and failure. The disease is mainly prevalent in Southeast Asian countries such as Japan and South Korea (9, 10). By 2023, China had reported more than 20,000 cases of SFTS (11), the highest number in the world. This disease has affected most provinces of China, and the cases were mainly concentrated in the eastern provinces such as Henan, Hubei, Shandong, Anhui, Liaoning, Jiangsu, and Zhejiang, especially in rural mountainous and hilly regions (12).

Previous research suggests that the prevalence of SFTS is influenced by a variety of environmental and social factors (12, 13). Environmental factors, especially meteorological factors including temperature, atmospheric pressure, precipitation, and relative humidity, not only affect the growth of ticks but also influence human activities, thus impacting the occurrence of SFTS (14, 15). Some studies have revealed a complex nonlinear relationship between meteorological factors and the incidence of SFTS (16, 17). SFTS in Jiangsu Province has exhibited an increasing trend in both incidence and spatial distribution, with the incidence rate increasing from 0.02 per 100,000 in 2011 to 0.14 per 100,000 in 2021 (18). Moreover, SFTS has a high mortality rate among middle-aged and older adult populations, and it has become a significant public health concern. Therefore, it is necessary to explore the epidemiological characteristics of SFTS and its relationship with environmental factors to inform strategies for targeted prevention and control efforts. This study intends to preliminarily analyze the epidemiological characteristics of SFTS in Jiangsu Province from 2014 to 2023, and to analyze the effects of meteorological factors on SFTS in the high prevalence areas of Jiangsu Province by using Distributed Lag Non-Linear Models (DLNM).

## 2 Materials and methods

### 2.1 Study area

**Figure 1:**
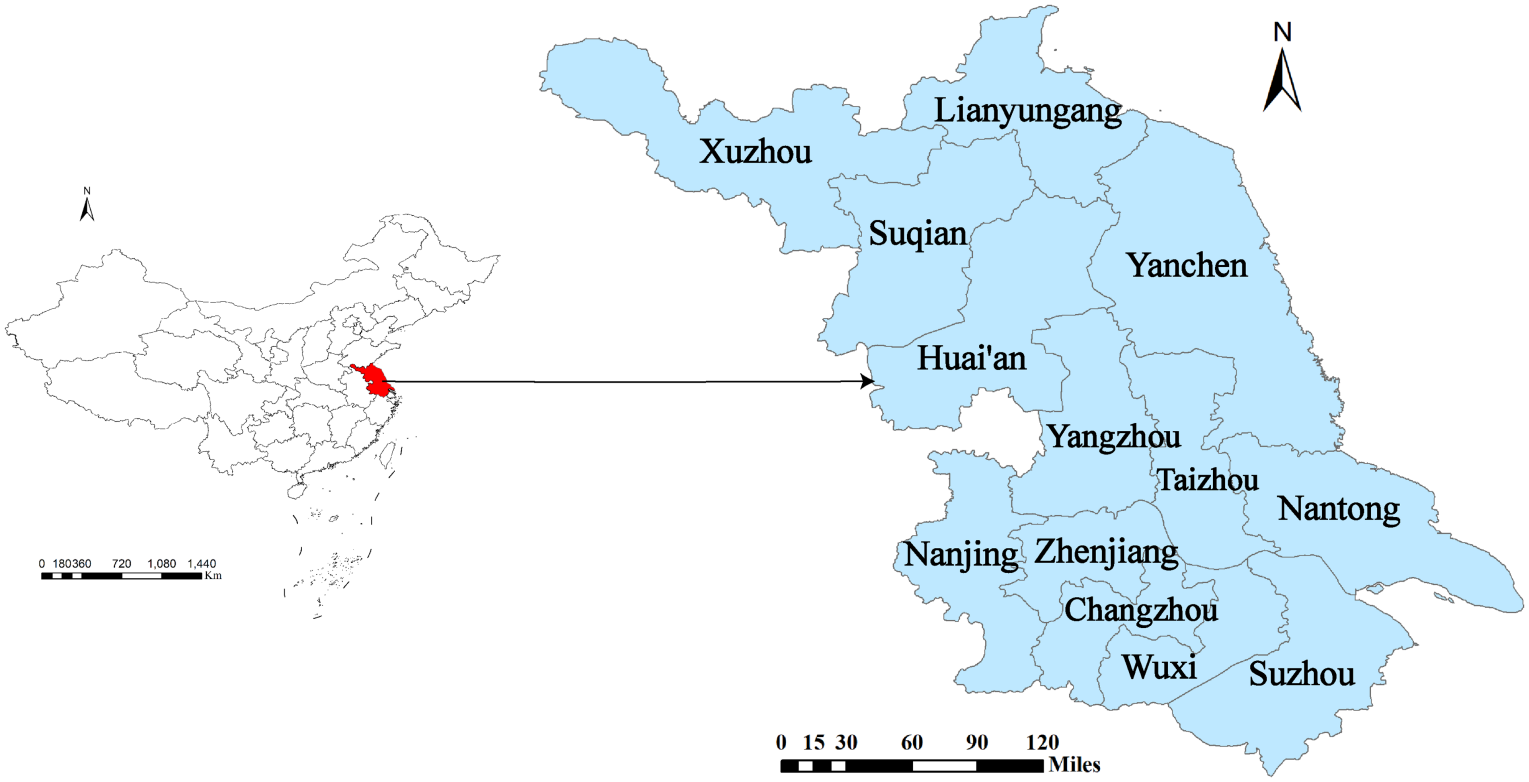
Location of Jiangsu Province in China and the administrative divisions of its 13 prefecture-level cities.

Jiangsu Province, with Nanjing as its capital, is located on the eastern coast of China. It spans both temperate and subtropical monsoon climate zones. It consists of 13 cities, while each city consists of several counties. It covers an area of 102,600 km^2^ and has a resident population of nearly 85 million (Figure 1).

### 2.2 Data collection

Case data in Jiangsu Province from January 2014 through December 2023 were sourced from the National Notifiable Disease Reporting Information System. The information of each case included residential address, age, gender, occupation, and date of illness onset. Meteorological data were obtained from the National Meteorological Science Data Sharing Service Platform,^1^ including average temperature (°C), average atmospheric pressure (hPa), 24-h precipitation (mm), average wind speed (m/s), relative humidity (%), and duration of sunshine (hours). Both case data and meteorological data were compiled on a weekly basis.

### 2.3 Joinpoint regression

Joinpoint 10.8 software was used to analyze the incidence trend of SFTS in Jiangsu Province from 2014 to 2023. Joinpoint regression models include linear models and log-linear models (19). The probability distribution of the number of SFTS cases was supposed to approximately follow a Poisson distribution. In this study, a log-linear model was selected, and the model expression was as follows:


$$E[y_{i}|x_{i}]=e^{\beta_{0}+\beta_{1}x+\delta_{1}{\left(x-\tau_{1}\right)}^{+}+\ldots +\delta_{k}{\left(x-\tau_{k}\right)}^{+}},$$

where *y_i_* (*i* = 1, 2, …, n) denotes the incidence rate of SFTS, *x_i_* (*i* = 1, 2, …, n) the year of illness onset. *β*_0_ represents the constant parameter, *β*_1_ represents the slope parameter or regression coefficient. *k* is the number of breakpoints, *δ_k_* represents the regression coefficient of the *k*th piecewise function, τ*_k_* is the *k*th unknown breakpoint, if (*x* - *τ_k_*) > 0, (*x* - *τ_k_*)^+^ = (*x* - *τ_k_*), otherwise, (*x* - *τ*_k_)^+^ = 0. The grid search method (GSM) was employed to determine the turning points in the incidence rates. The annual percentage changes (APC) before and after the turning points were computed, and parameters were validated through Monte Carlo permutation tests.

### 2.4 Distributed lag non-linear model (DLNM)

Spearman’s correlation analysis was conducted to identify the meteorological factors significantly correlated with the incidence of SFTS. These factors were then integrated into DLNM to precisely analyze the exposure-response relationship and lag effects between the meteorological factors and SFTS in high-incidence cities in Jiangsu Province. These cities represent stable high-incidence foci of SFTS in Jiangsu Province, which facilitates a clearer identification of associations with meteorological factors. Therefore, DLNM was applied to quantify the exposure-lag-response relationships between meteorological variables and SFTS incidence in these settings. To avoid the influence of excessive dispersion, the analysis employed a quasi-Poisson distribution with a log-link function to formulate the regression model. The model is as follows:


$$\log(Y_{w})=\alpha +\mathrm{cb}(X,\mathrm{lag})+\sum \mathrm{ns}(Z_{i},\mathrm{df})+\mathrm{ns}(\text{Time},\mathrm{df}\times \text{year}),$$

where *Y_w_* represents the weekly number of cases; *α* is the intercept of the model; *cb*() represents the cross-basis matrix, and *X* is the primary meteorological factor being evaluated. The exposure-response relationship is modeled using a quadratic B-spline function, and the *lag* parameter is represented using a natural cubic spline function (*ns*). Both the exposure-response and lag-response dimensions were assigned 3 degrees of freedom (*df*), in accordance with previous studies (20), to adequately capture nonlinear relationships. The maximum lag (in weeks) was determined by selecting the value that minimized the quasi-Akaike Information Criterion (Q-AIC) in the quasi-Poisson model (20). *Z_i_* represents other meteorological factors related to X and is used to control the confounding effects. *Time* is a time series variable, assigned values 1, 2, 3, … 523, to reflect seasonality and long-term trends.

### 2.5 Statistical analysis

ArcGIS 10.7 software was employed to graphically represent the geographical distribution of cases. R software version 4.2.3 was utilized to analyze the overview and epidemiological characteristics of SFTS in Jiangsu Province from 2014 to 2023. Especially, R package “dlnm” was utilized to fit the DLNM model. All statistical tests were two-sided, and a *p*-value < 0.05 was deemed as statistically significant.

## 3 Results

### 3.1 Epidemiological characteristics of SFTS

From 2014 to 2023, a total of 914 cases of SFTS were reported in Jiangsu Province, with an average annual incidence rate of 0.11 per 100,000 population. Among all reported cases, 68 were fatal, resulting in an average case fatality rate of 7.44%. Among the reported cases, there were 468 males and 446 females, reaching a male-to-female ratio of 1.05:1. The median ages of male and female cases were 65 years [interquartile range (IQR): 54–71; standard deviation (SD): 12.8] and 67 years (IQR: 57–73; SD: 11.8), respectively. Cases aged 50–79 years accounted for 83.48% (763/914) of the total cases (Table 1). A statistically significant difference in the ages of onset was observed between genders (*p* < 0.05). Most affected individuals were farmers (69.14%, 632/914), followed by houseworkers, retirees, or the unemployed (22.54%, 206/914).

**Table 1:** Population characteristics of SFTS in Jiangsu Province from 2014 to 2023.

Age group	Cases		Incidence (per 100,000)	
	Male	Female	Male	Female
~20	2	2	0.02	0.03
20–29	7	4	0.15	0.10
30–39	18	8	0.25	0.12
40–49	38	28	0.66	0.49
50–59	104	105	1.37	1.35
60–69	153	131	2.77	2.51
70–79	127	143	3.92	4.16
80~	19	25	1.47	1.43
Total	468	446	1.08	1.06

**Figure 2:**
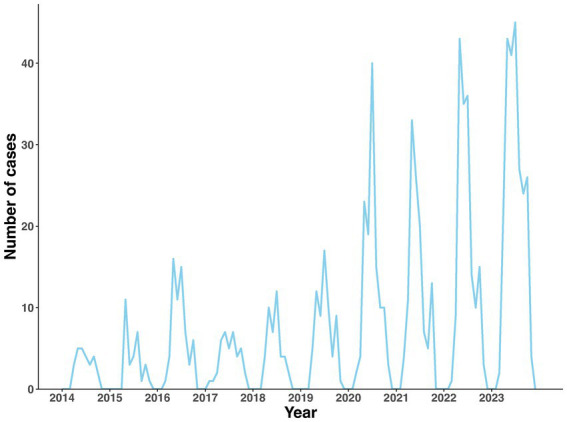
Temporal trends of monthly reported SFTS incidence in Jiangsu Province from 2014 to 2023.

The results of the Joinpoint regression show that the trend of SFTS incidence adhered to a logarithmic-linear model, with no turning points detected. Over the decade, the incidence demonstrated an increasing trend, with an APC of 26.68% (95% CI: 19.22–34.60%, *p* < 0.001) (Supplementary Figure S1). The incidence exhibited marked seasonality. More than 97% of total cases occurred between April and October, with an incidence peak observed between May and July. No cases were reported in January, February, and December (Figure 2).

**Figure 3:**
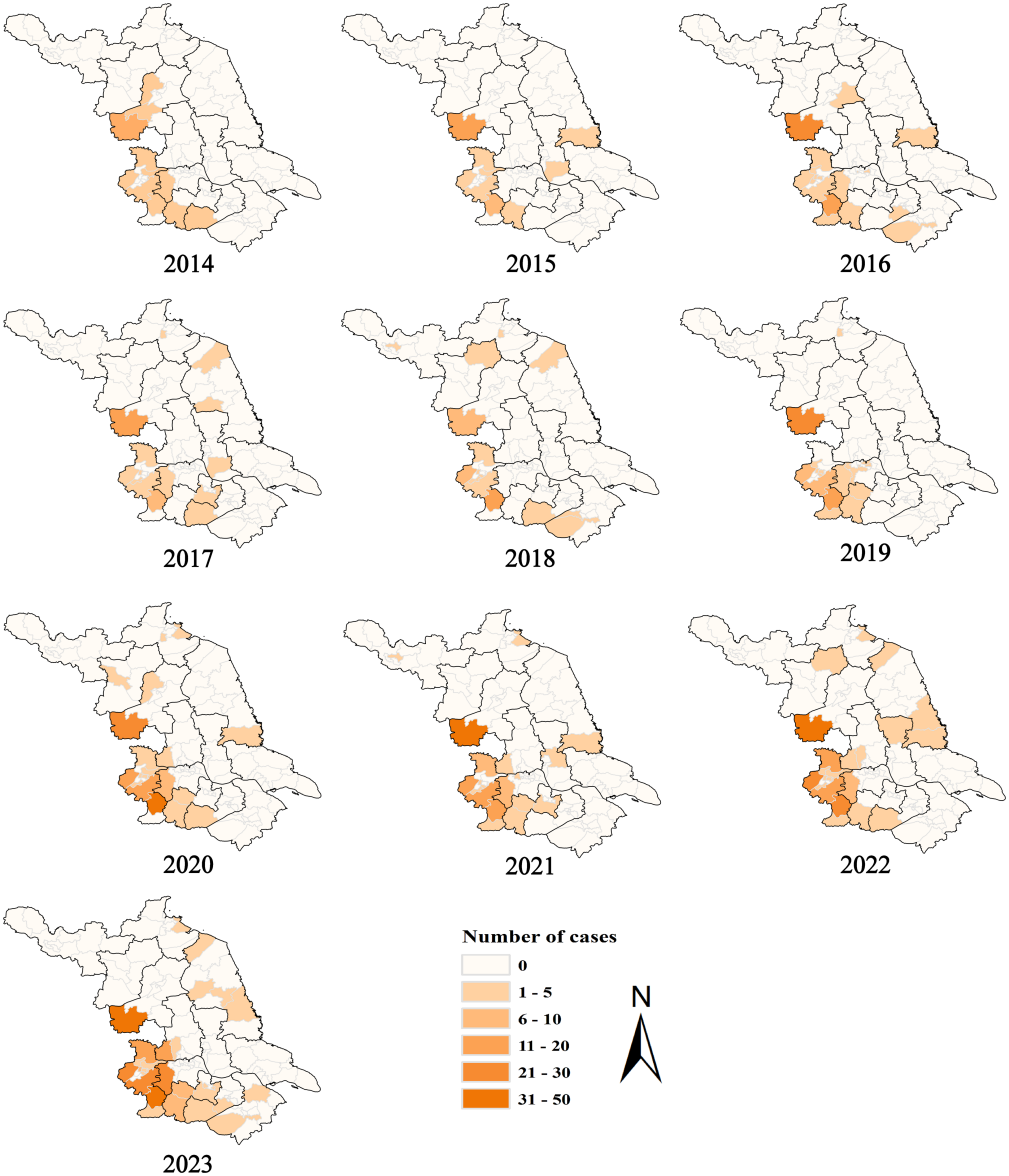
Spatial distribution of SFTS cases in Jiangsu Province from 2014 to 2023.

During 2014–2023, SFTS cases were identified in 49 counties in Jiangsu Province, with the number of affected counties rising from 11 in 2014 to 28 in 2023 (Figure 3). Among the 13 cities, only Nantong City did not report any cases of SFTS. Notably, Nanjing and Huai’an were the major endemic regions, with 489 and 236 cases, respectively, together accounting for 79.32% of the total cases in the province.

In addition, the median interval from symptom onset to diagnosis exhibited a decreasing trend over time. The median diagnostic delay was 9 days in 2014, peaked at 13 days in 2016, and gradually declined to 5 days by 2023. The overall median delay across the study period was 7 days (Supplementary Figure S2).

Logistic regression analysis, using age as a continuous variable, indicated that each additional year of age was associated with a 5.7% increase in the odds of death (OR = 1.06, 95% CI: 1.03–1.09, *p* < 0.001). Stratified analysis further demonstrated that no deaths occurred among individuals under 50 years of age, while the case fatality rate was 5.26% in individuals aged 50–59 years, 6.34% in 60–69 years, 12.20% in 70–79 years, and 13.67% in those aged 80 years and above.

### 3.2 Relationship between SFTS and meteorological factors

As the major endemic regions of SFTS in Jiangsu Province, Nanjing and Huai’an were selected as study sites to investigate the association between SFTS incidence and meteorological factors. Compared with Huai’an, Nanjing exhibited slightly higher average temperature and cumulative precipitation, while Huai’an had longer sunshine duration. Relative humidity and atmospheric pressure were similar between the two cities. Notably, wind speed in Nanjing was nearly twice that in Huai’an (Supplementary Tables S1 and S2). Spearman correlation analysis revealed a moderate association between weekly average temperature and SFTS incidence in both Nanjing and Huai’an. Weak correlations were observed for precipitation and sunshine duration in both cities. A weak correlation with relative humidity was identified only in Huai’an. Atmospheric pressure was negatively correlated with SFTS incidence in both locations (Tables 2, 3). In both cities, weekly average temperature was strongly correlated with atmospheric pressure (| r_s_ | > 0.9), and moderate correlations were also observed among temperature, precipitation, sunshine duration, and relative humidity. DLNM models were independently established for Nanjing and Huai’an. To avoid multicollinearity, weekly average temperature-rather than atmospheric pressure-was incorporated into the models to evaluate the exposure-response and lag effects on SFTS incidence. Other meteorological variables correlated with temperature were treated as confounding factors in the models.

**Table 2:** Spearman correlation analysis of SFTS incidence and meteorological factors in Nanjing from 2014 to 2023.

Variable (week)	Cases	Temperature	Precipitation	Sunshine duration	Relative humidity	Atmospheric pressure
Temperature	0.57*					
Precipitation	0.17*	0.36*				
Sunshine duration	0.18*	0.34*	−0.39*			
Relative humidity	0.08	0.19*	0.59*	−0.67*		
Atmospheric pressure	−0.56*	−0.94*	−0.45*	−0.25*	−0.22*	
Wind speed	−0.08	0.02	0.16*	−0.03	0.02	−0.06

*Means *p* < 0.05.

**Table 3:** Spearman correlation analysis of SFTS incidence and meteorological factors in Huai’an from 2014 to 2023.

Variable (week)	Cases	Temperature	Precipitation	Sunshine duration	Relative humidity	Atmospheric pressure
Temperature	0.46*					
Precipitation	0.21*	0.51*				
Sunshine duration	0.15*	0.34*	−0.17*			
Relative humidity	0.12*	0.42*	0.56*	−0.25*		
Atmospheric pressure	−0.44*	−0.94*	−0.54*	−0.29*	−0.38*	
Wind speed	0.01	−0.02	0.27*	0.07	−0.08	−0.09

*Means *p* < 0.05.

**Figure 4:**
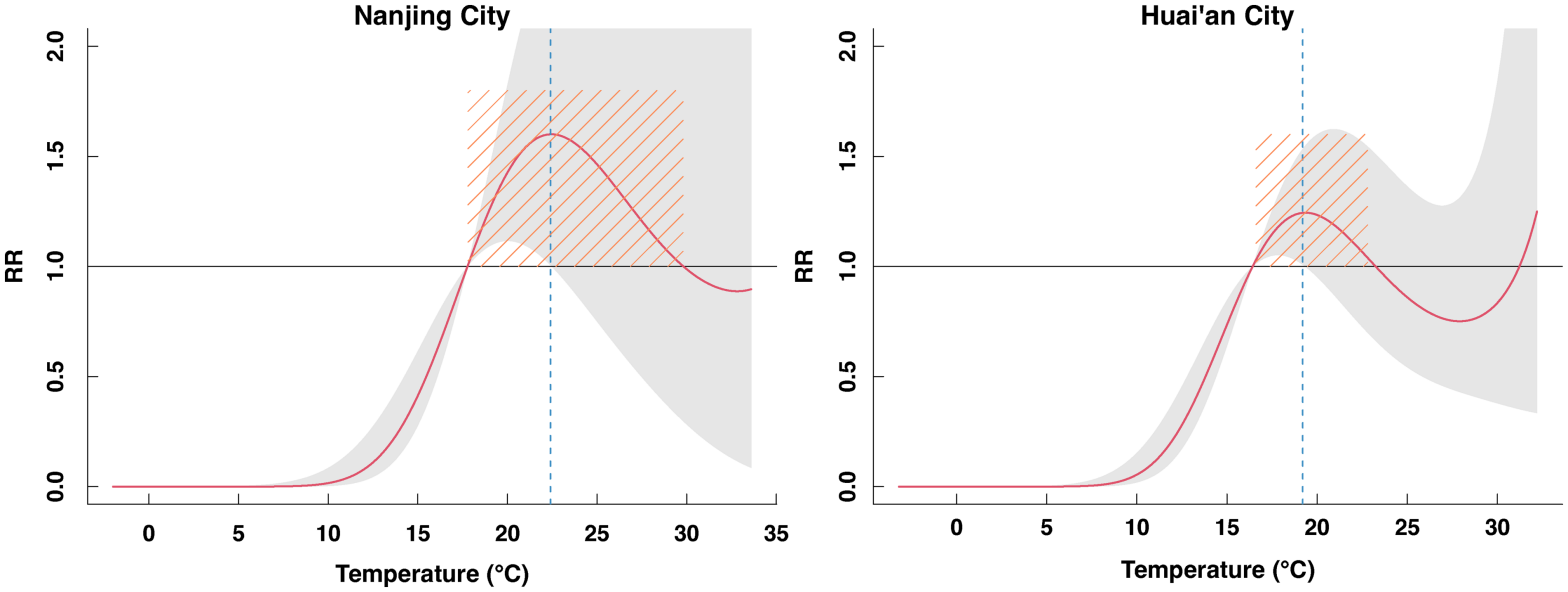
Cumulative relative risk (RR) curves for SFTS incidence associated with temperature in Nanjing and Huai’an, based on DLNM analysis.

The results suggested that the overall cumulative effect curve of the weekly average temperature on SFTS incidence in Nanjing exhibits an inverted “V” shape. At a lag of 12 weeks, the relative risk (RR) increased with rising weekly average temperatures. The RR peaked at a weekly average temperature of 22.4 °C (RR = 1.60, 95% CI: 1.01–2.55). The weekly average temperature ranged from 17.8 °C to 29.8 °C, with RR greater than 1, indicating that this temperature range promoted the occurrence of SFTS. In Huai’an, the inflection point for weekly average temperature occurred at 19.2 °C (RR = 1.23, 95% CI: 1.00–1.52). The temperature range of promoting SFTS was 16.6–22.8 °C (Figure 4).

**Figure 5:**
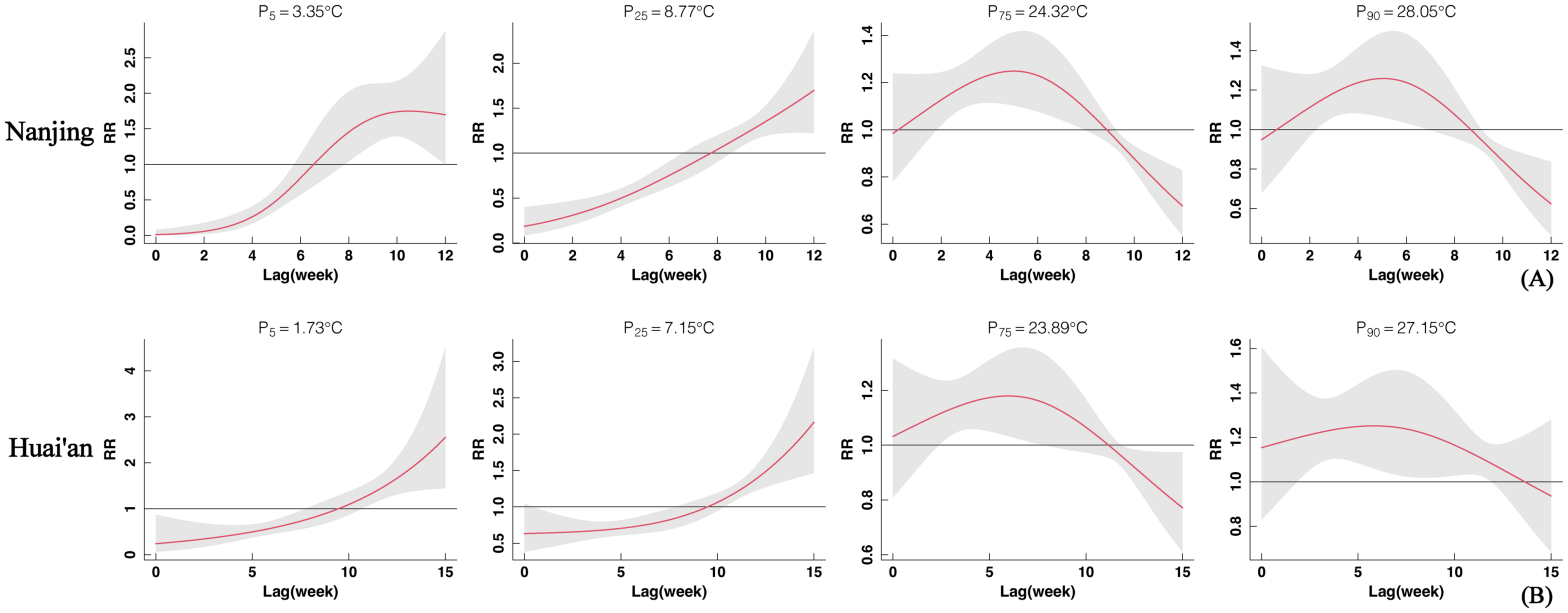
Distributed lag effects of temperature on SFTS incidence risk in Nanjing **(A)** and Huai’an **(B)**, based on DLNM analysis.

In Nanjing, at lower temperatures of 3.35 °C and 8.77 °C, the lag effect distribution curves exhibited a clear upward trend. At a lag of 0 weeks, the risk of SFTS incidence was lowest, with RR values of 0.01 (95% CI: 0.001–0.08) and 0.19 (95% CI: 0.09–0.40), respectively, indicating a significant protective effect for temperatures within a 0–6 weeks lag. Conversely, at higher temperatures of 24.32 °C and 28.05 °C, the lag effect curves initially increased and then decreased over time, with significant risk effects observed between 2 and 7 weeks lag (Figure 5A). In Huai’an, the relationship between specific temperatures and SFTS incidence exhibited similar trends as the lag weeks increased (Figure 5B).

## 4 Discussion

This study analyzed the SFTS surveillance data in Jiangsu Province from 2014 to 2023. The annual incidence rates exhibited an overall upward trend, mainly attributed to increased national attention on SFTS in recent years, along with strengthened surveillance and improved diagnostic capabilities (12). Concurrently, the median time from symptom onset to diagnosis showed a decreasing trend, further reflecting improvements in early case identification. The incidence also presented remarkable seasonality, with higher incidence rates during the summer and autumn seasons. Tick reproduction and growth primarily occur in the summer (21), coinciding with the peak incidence period of SFTS. Spatially, cases were primarily concentrated in Nanjing and Huai’an, with a progressive geographic expansion to surrounding areas. This spatial pattern reflects the typical clustering nature of SFTS transmission. The continuous increase in SFTS incidence and the gradual expansion of epidemic areas may be closely linked to the natural dispersal of ticks and their host animals. Middle-aged and older adults, particularly agricultural workers, form the primary demographic groups affected by SFTS. In rural families, most older adults engage in farming and are frequently exposed to ticks. Moreover, older adults exhibit higher case fatality rates, indicating a dual burden of higher incidence and mortality in this population. As a result, a higher proportion of SFTS cases is observed among farmers and older adults in rural communities (22). These epidemiological characteristics share certain commonalities across eastern China, including neighboring provinces such as Shandong, Zhejiang, and Anhui (23–25).

Prior research has demonstrated that meteorological factors can influence the occurrence and spread of infectious diseases (26, 27). Xiang et al. used the DLNM method to analyze the impact of meteorological factors on the incidence of dengue fever and its lag effects. Their results indicated an inverse “U”-shaped nonlinear relationship between temperature, relative humidity, and wind speed, and the number of dengue fever cases (28). Yu et al. (29) applied the DLNM to demonstrate that extreme temperatures, high precipitation, and low ozone levels increase the risk of hand, foot, and mouth disease. They observed that the effects of high temperatures were more pronounced and persisted longer than those of low temperatures. Tao et al. utilized a niche model to analyze the significant correlation between tick density, precipitation in the wettest month, average temperature, altitude, and the normalized difference vegetation index (NDVI) with the spatial distribution of SFTS (30). As ticks serve as vectors for SFTSV, their growth and development are influenced by meteorological factors, thereby indirectly affecting the incidence of SFTS (31). According to the results of the DLNM, this study identified significant correlations between the temperature and the incidence of SFTS in the high incidence areas of Jiangsu Province, revealing pronounced nonlinear and lag effects.

Zhang et al. (32) reported that the highest temperatures during the hottest months promote the occurrence of SFTS in Jiangsu Province. In this study, the relationship between temperature and the cumulative effect on SFTS, as represented by RR values, initially increases and then gradually decreases, forming an inverted “V”-shaped curve. The temperature inflection points in Nanjing and Huai’an were 22.4 °C and 19.2 °C, respectively. The lag effects of temperature on SFTS are similar to those reported in Sun et al.’s (33) research. The peak activity period for tick populations typically occurs in spring and early summer when temperatures are around 20 °C (34). Within this temperature range, the survival capacity of ticks is significantly enhanced, enabling an expansion of their habitat range. Certain developmental stages of ticks, such as nymphs, are particularly sensitive to temperature fluctuations; disruptions at these stages can lead to reductions in overall tick population density. Furthermore, moderate temperatures (~20 °C) are conducive to outdoor activities among humans, who frequently visit parks, forests, and other green spaces, thereby increasing their risk of exposure to ticks and subsequent tick-borne diseases. Additionally, suitable temperatures around 20 °C favor outdoor activity and grazing of host animals such as cattle, sheep, and dogs, elevating the probability of these animals being bitten by ticks and indirectly increasing the risk of human infection via these animal hosts. In suitable high-temperature environments, ticks become more active and rapidly progress through various stages of their lifecycle. However, sustained high temperatures may eventually cause ticks to perish due to dehydration and overheating. Conversely, at lower temperatures, the lag effect of temperature exhibits a protective role against SFTS incidence, although this effect gradually diminishes with increasing lag periods. In cold environments, ticks exhibit decreased metabolic rates, restricting their activity and reproductive capacity; extremely low temperatures may even threaten their survival, reducing their population density (35). Moreover, as weather cools, humans tend to wear more clothing and reduce outdoor activities, further decreasing the risk of tick bites.

There are differences in average temperatures between Nanjing and Huai’an, which may also contribute to the variations observed in the lag effects and inflection points of temperature between the two cities. Other studies have shown that, in addition to temperature and atmospheric pressure, meteorological factors such as relative humidity, cumulative precipitation, and wind speed also exhibit lag effects on the incidence of SFTS (22, 36). These inconsistencies suggest that the impact of meteorological factors on SFTS transmission may vary by region, likely due to differences in ecological environments, human activity patterns, and local vector-host dynamics (37).

This study has certain limitations. Firstly, the use of population-level data may introduce ecological bias when inferring individual-level associations. Secondly, there were likely cases of missed diagnosis in the early years of surveillance due to inadequate diagnostic capacity in some medical institutions, which may have resulted in underestimation of SFTS incidence in Jiangsu Province and introduced potential reporting bias. Thirdly, the model did not incorporate important variables such as socioeconomic factors, tick density, or host animal data, which may limit our understanding of the environmental and ecological determinants of SFTS transmission. Lastly, the meteorological data used in the analysis were aggregated at the prefecture level rather than the more granular county level, which could have attenuated the estimated lagged associations between meteorological factors and disease occurrence due to reduced spatial resolution. Therefore, future research should aim to incorporate additional influencing variables, improve diagnostic data quality, and enhance spatial resolution to enable more comprehensive and accurate risk assessments.

## 5 Conclusion

In summary, the incidence rate of SFTS in Jiangsu Province demonstrates an increasing trend. A nonlinear relationship exists between weekly average temperature and the risk of SFTS, characterized by persistent and lagged effects on the disease incidence. This result underscores the need for targeted public awareness and preventive interventions among middle-aged and older adults farmers as the average temperature approaches 15 °C during the transition from spring to summer. It is crucial to strengthen the understanding of high-risk groups on the transmission routes of SFTS through health education, and train them on the correct use of protective clothing (long sleeves, pants tucked into boots), gloves, and effective repellents specifically during agricultural activities in tick-prone areas. In addition, environmental management should be strengthened, and feasible strategies and measures should be adopted to reduce tick habitats around farmland and residential areas.

## Data Availability

The datasets presented in this article are not readily available because SFTS data underlying the results presented in the study cannot be shared publicly because of the limitation of data availability in the data management rule of the Jiangsu Center for Disease Control and Prevention. Requests to access the datasets should be directed to CB, bao2000_cn@163.com.

## Footnotes

^1^ http://data.cma.cn/

## Supplementary material

SUPPLEMENTARY FIGURE S1 Trend of the incidence of SFTS between 2014 and 2023 shown by the joinpoint regression.

SUPPLEMENTARY FIGURE S2 Trend in the average time from symptom onset to diagnosis of SFTS in Jiangsu Province from 2014 to 2023.
