# Supplementary figures and images for: Epidemiological characteristics of severe fever with thrombocytopenia syndrome and the relationship with meteorological factors in Jiangsu Province, China
Source: Front Public Health. 2025 Sep 1;13:1662670. doi: 10.3389/fpubh.2025.1662670 (PMC12434120; doi:10.3389/fpubh.2025.1662670)

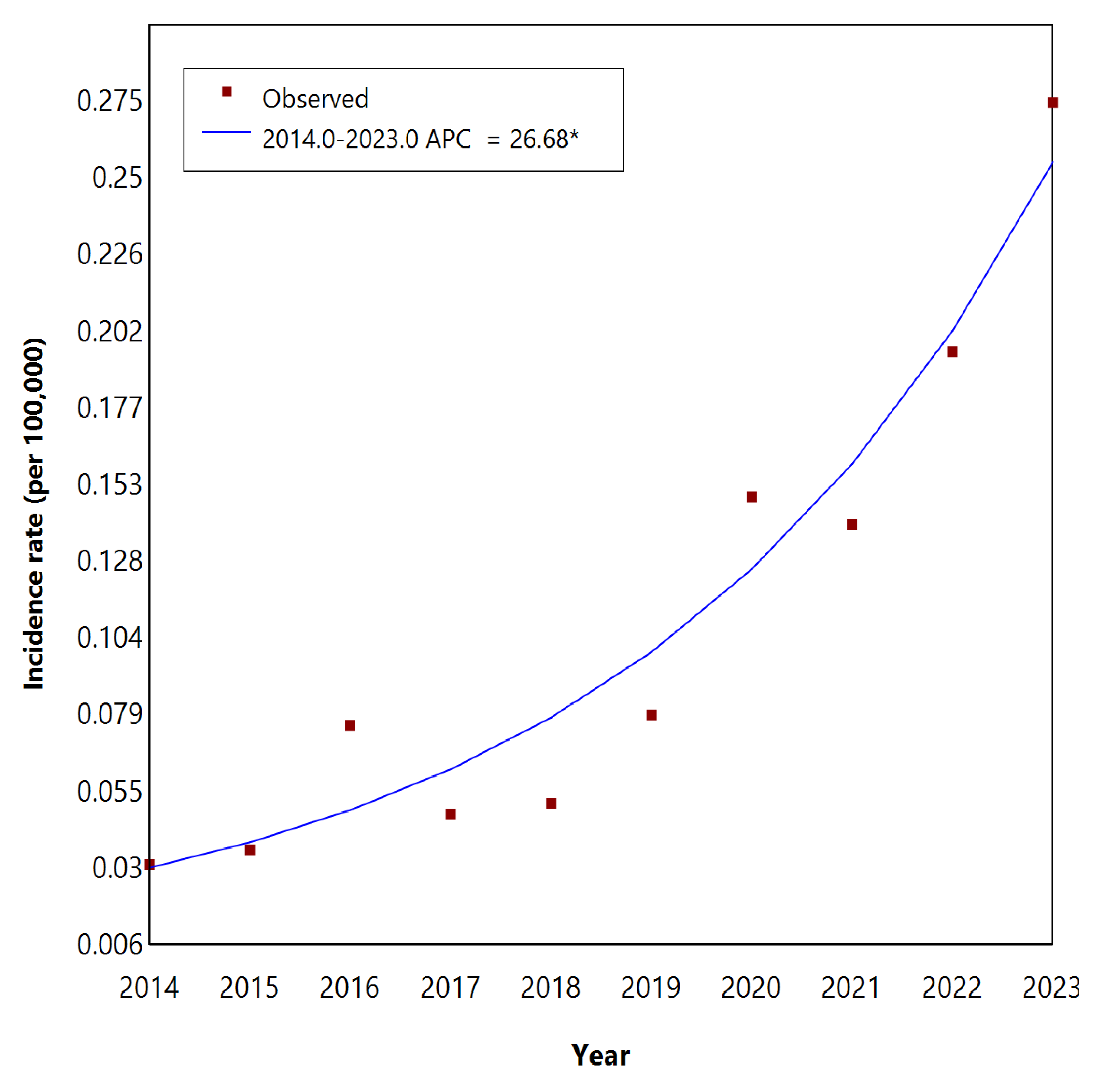

Supplement: SUPPLEMENTARY FIGURE S1 — Trend of the incidence of SFTS between 2014 and 2023 shown by the joinpoint regression. [file Image_1.tif]

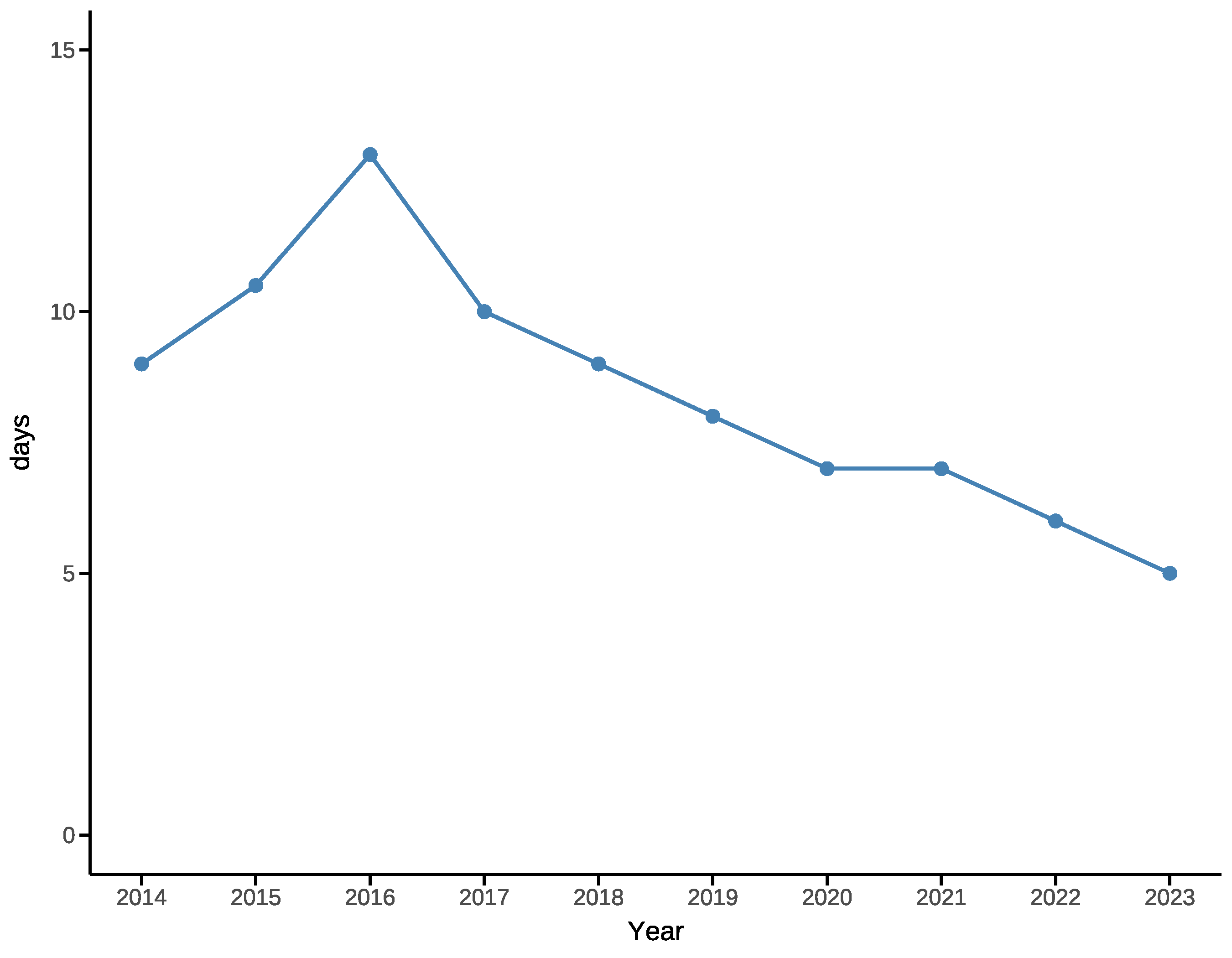

Supplement: SUPPLEMENTARY FIGURE S2 — Trend in the average time from symptom onset to diagnosis of SFTS in Jiangsu Province from 2014 to 2023. [file Image_2.tif]
