# Supplementary material for: Epidemiological characteristics of severe fever with thrombocytopenia syndrome and the relationship with meteorological factors in Jiangsu Province, China
Source: Front Public Health. 2025 Sep 1;13:1662670. doi: 10.3389/fpubh.2025.1662670 (PMC12434120; doi:10.3389/fpubh.2025.1662670)
Supplement: Supplementary file 3 [file Supplementary_file_1.docx]

**Table S1** Descriptive information of the weekly meteorological factors in Nanjing from 2014 to 2023.

| Variable（Week） | Mean | SD | Minimum | *P_25_* | *P_50_* | *P_75_* | Maximum |
| --- | --- | --- | --- | --- | --- | --- | --- |
| Temperature | 16.89 | 8.80 | -2.13 | 8.77 | 17.77 | 24.32 | 33.64 |
| Precipitation | 24.06 | 26.94 | 0.00 | 5.63 | 16.39 | 33.37 | 170.97 |
| Sunshine duration | 4.99 | 2.36 | 0.00 | 3.24 | 5.01 | 6.68 | 11.36 |
| Relative humidity | 74.83 | 9.19 | 48.57 | 68.46 | 75.49 | 81.95 | 94.14 |
| Atmospheric pressure | 1016.73 | 8.95 | 995.44 | 1008.35 | 1017.16 | 1024.09 | 1038.06 |
| Wind speed | 4.20 | 1.48 | 1.44 | 2.69 | 4.51 | 5.21 | 8.71 |

Note: SD is standard deviation; *P_x_* means *x*th percentile.

**Table S2** Descriptive information of the weekly meteorological factors in Huai'an from 2014 to 2023.

| Variable（Week） | Mean | SD | Minimum | *P_25_* | *P_50_* | *P_75_* | Maximum |
| --- | --- | --- | --- | --- | --- | --- | --- |
| Temperature | 15.69 | 9.12 | -3.30 | 7.15 | 16.42 | 23.89 | 32.20 |
| Precipitation | 20.50 | 23.56 | 0.00 | 3.32 | 12.24 | 29.42 | 137.29 |
| Sunshine duration | 6.25 | 4.55 | -0.59 | 3.68 | 5.50 | 7.17 | 29.24 |
| Relative humidity | 74.81 | 9.50 | 50.82 | 67.29 | 75.37 | 82.87 | 93.03 |
| Atmospheric pressure | 1017.01 | 9.03 | 996.66 | 1008.74 | 1017.30 | 1024.54 | 1038.33 |
| Wind speed | 2.19 | 1.06 | 0.00 | 1.71 | 2.07 | 2.51 | 13.55 |

Note: SD is standard deviation; *P_x_* means *x*th percentile.
